# A Geographic Assessment of the Global Scope for Rewilding with Wild-Living Horses (*Equus ferus*)

**DOI:** 10.1371/journal.pone.0132359

**Published:** 2015-07-15

**Authors:** Pernille Johansen Naundrup, Jens-Christian Svenning

**Affiliations:** Section for Ecoinformatics and Biodiversity, Department of Bioscience, Aarhus University, Aarhus C, Denmark; Université de Sherbrooke, CANADA

## Abstract

Megafaunas worldwide have been decimated during the late Quaternary. Many extirpated species were keystone species, and their loss likely has had large effects on ecosystems. Therefore, it is increasingly considered how megafaunas can be restored. The horse (*Equus ferus*) is highly relevant in this context as it was once extremely widespread and, despite severe range contraction, survives in the form of domestic, feral, and originally wild horses. Further, it is a functionally important species, notably due to its ability to graze coarse, abrasive grasses. Here, we used species distribution modelling to link locations of wild-living *E*. *ferus* populations to climate to estimate climatically suitable areas for wild-living *E*. *ferus*. These models were combined with habitat information and past and present distributions of equid species to identify areas suitable for rewilding with *E*. *ferus*. Mean temperature in the coldest quarter, precipitation in the coldest quarter, and precipitation in the driest quarter emerged as the best climatic predictors. The distribution models estimated the climate to be suitable in large parts of the Americas, Eurasia, Africa, and Australia and, combined with habitat mapping, revealed large areas to be suitable for rewilding with horses within its former range, including up to 1.5 million ha within five major rewilding areas in Europe. The widespread occurrence of suitable climates and habitats within *E*. *ferus*’ former range together with its important functions cause it to be a key candidate for rewilding in large parts of the world. Successful re-establishment of wild-living horse populations will require handling the complexity of human–horse relations, for example, potential conflicts with ranchers and other agriculturalists or with other conservation aims, perception as a non-native invasive species in some regions, and coverage by legislation for domestic animals.

## Introduction

During the late Quaternary, megafaunas worldwide have been decimated [1]. It is increasingly clear that humans have played a key role in these losses [2–4], and human-driven megafauna losses are still ongoing, e.g., with a great proportion of extant perissodactyl species now extinct in a large part of their historical range and/or declining and severely threatened (14 out 16 species) [5]. Large herbivores and carnivores can have large impacts on ecosystems, often serving as keystone species and ecosystem engineers [6, 7], and the megafauna losses have had profound effects on ecosystems [8–10]. Reintroduction of extirpated species or functional types of high ecological importance to restore self-managing functional, biodiverse ecosystems (rewilding) is increasingly being discussed and implemented [11]. Rewilding is being implemented in various ways, notably at varying spatial scales and with varying degrees of ongoing human interventions [11]. Scale and potential human-wildlife conflicts are some of the factors that require consideration, as do ecological effects when reintroducing species that have been absent for thousands of years [12–16].

The horse *(Equus ferus*) is among the species with particularly high relevancy for rewilding [17]. The species has experienced a massive range collapse since the Late Pleistocene, i.e., in terms of originally wild populations. However, one originally wild subspecies (*E*. *f*. *przewalskii*) has survived, and much genetic diversity has been preserved in the domesticated forms [18–21]. The species was extremely widespread and common during the Middle and Late Pleistocene, with a distribution that covered most of Eurasia and northern Africa as well as North and South America [22–33]. It evolved in North America 1.1–1.2 Ma [26] and spread via the Beringia land bridge and the Isthmus of Panama to Eurasia and South America some 0.9–0.8 Ma [26, 34] and to Africa in the Late Pleistocene [26]. Further, *E*. *ferus* was just the latest of a more or less long line of grazing equids in these regions [35, 36]. In the Americas, *E*. *ferus* went extinct during the latest Pleistocene or early Holocene [26, 33, 34, 37], whereas it remained widespread in the wild in Eurasia until the late Holocene [38, 39]. *Equus ferus* was domesticated ca. 3600 BC [40]. The last originally wild populations disappeared from Eastern Europe and the southern parts of Russia during the last few hundred years [39], whereas the subspecies *E*. *f*. *przewalskii* survived until 1969 in the wild in Central Asia. It has been reintroduced in the wild in the region again from 1992 onwards [41]. Anthropogenic factors are clearly the cause of the extinction of wild *E*. *ferus* in Eurasia, notably hunting and domestication [26, 40]. The cause of extinction in the Americas is less clear, but the evidence there also point to humans rather than climate being the cause [37, 42–46], notably when examined in the context of broader megafauna extinctions [8]. A genetic study of domestic *E*. *ferus* and the Asiatic wild horse (*E*. *f*. *przewalskii*) show that the domestic *E*. *ferus* harbour genetic diversity from 17 haplogroups, but not the haplogroup found in *E*. *f*. *przewalskii*, indicating that multiple maternity lines throughout Eurasia underwent domestication [21].

Despite the historical range collapse, wild-living horses are today found today in many parts of the world. In addition to the few reintroduced populations of *E*. *f*. *przewalskii* in Mongolia [41, 47], feral populations are found on all continents, except Antarctica [47]. Australia and New Zealand harbour large populations, and wild-living *E*. *ferus* is often considered an invasive pest there as the species was introduced by humans only 200 years ago [48–51] and the regions have never harboured native horses. North America likewise harbours large feral populations, and feral horses are there perceived either as an iconic native or semi-native species [52] or as a non-indigenous invasive species [53]. Several primitive or back-bred breeds exist, and some are already being used in rewilding projects in Europe [54].

**Fig 1:**
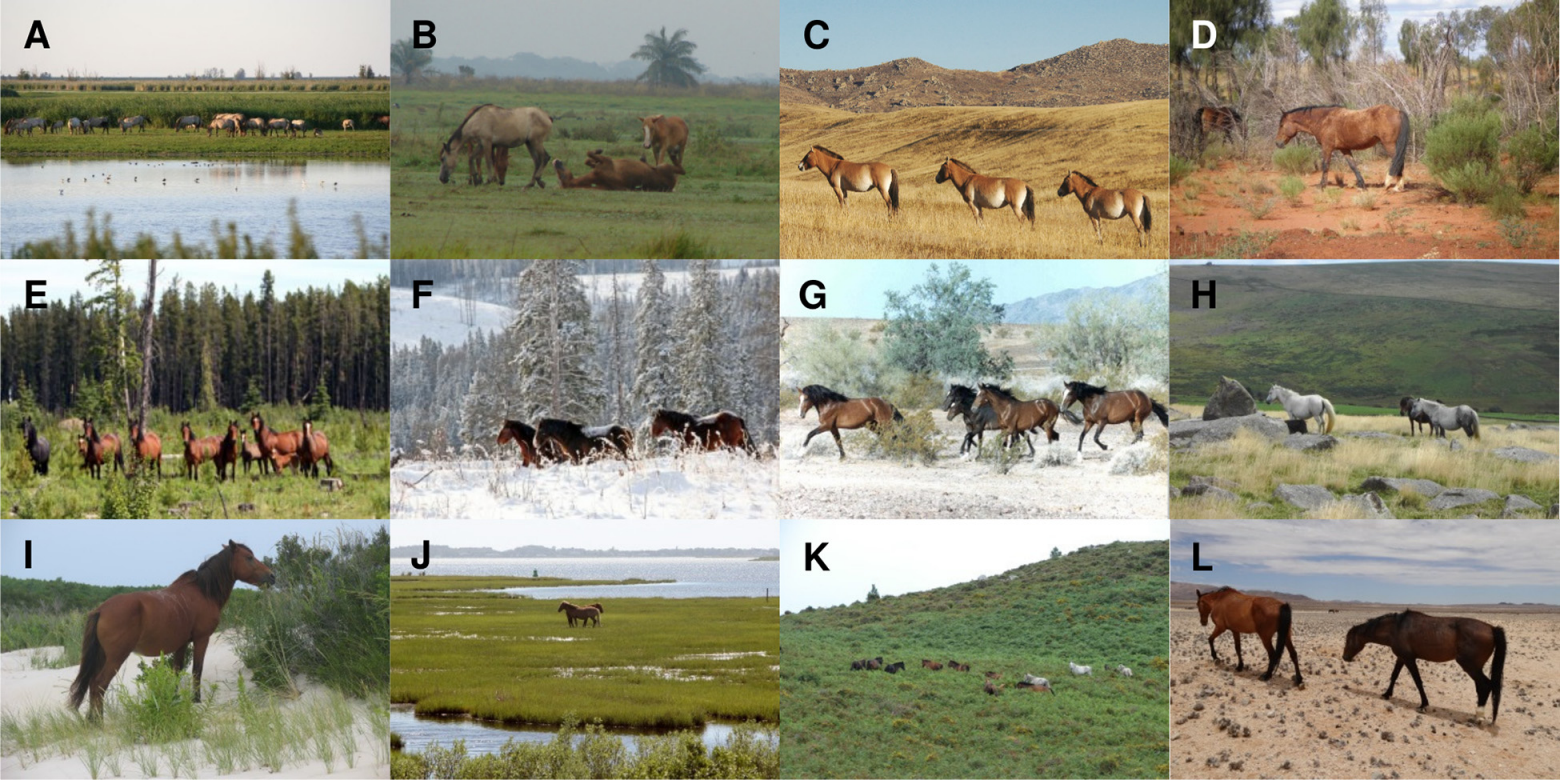
Feral *E*. *ferus* in a range of different habitats. Feral *E*. *ferus* inhabit areas worldwide with a wide range of habitats and climates, including Oostvaardersplassen, the Netherlands (**A**) (Credit: Eva Maria Kintzel and I Van Stokkum); tropical wet and dry seasons in Los Llanos, Venezuela (**B**) (Credit: Victor Ros Pueo); the Mongolian steppe in Hustai National Park, Mongolia (**C**) (Credit: Usukhjargal Dorj, Hustai National Park); the deserts of central Australia, western North America and Namibia (**D**; **G**; **L**) (Credit: Pernille J. Naundrup; Bureau of Land Management, USA; Telane Greyling); logged forests and snow covered winters in Alberta, Canada (**E-F**) (Credit: Bob Henderson); moorlands in Dartmoor, England (**H**) (Credit: Mark Robinson); feeding in the sand dunes and saltmarshes at Assateague Island, Maryland and Virginia, USA (**I-J**) (Credit: National Park Services, USA; Fritz Geller-Grimm, CC BY-SA 2.5); and in the mountains of Galicia, Spain (**K**) (Credit: Victor Ros Pueo).

Rewilding emphasises species reintroductions to restore ecological function [11, 12, 17], and *E*. *ferus* is clearly relevant in this perspective [17]. Large herbivores can have profound effects on their habitats. Different species have different effects on an ecosystem due to differences in morphology and ecology. Due to the widespread former distribution of *E*. *ferus* and earlier grazing equids, grassland biota in much of the world must have evolved and/or persisted under the influence of grazing by horses for millions of years [35, 36]. Being a selective grazer, *E*. *ferus* selects preferred grasses, sedges and herbs, including coarse, highly abrasive grasses [55], creating a mosaic of high and low vegetation in grasslands [17, 56]. This creates a more diverse habitat for invertebrates, small vertebrates and herbaceous plants. Other behaviours, such as its movement, also have ecological effects, opening up the vegetation and thereby promoting disturbance-dependent plant species [56]. In comparison with cattle and bison, two other species used for rewilding in Europe, *E*. *ferus* is more selective, feeding more on grasses and less on browse, may survive feeding on coarse, abrasive grasses, and bites the vegetation much closer to the ground [56]. Studies of feral horses demonstrate that they can have important effects on vegetation structure, biodiversity and ecosystem processes such as productivity and wetland sedimentation. [53, 57, 58]. *E*. *ferus* inhabits a wide variety of open habitats such as grasslands, steppe, shrub and desert [41, 49, 59–77] and can also be found in wetlands, marshes, heathlands, and woodlands [49, 53, 66, 67, 71, 77–79] (Fig 1).

Although *E*. *ferus* is an obvious candidate for rewilding in many areas, no study has hitherto assessed the general occurrence of ecologically suitable areas. Species distribution models (SDM) are important tools in conservation management [80–84] as they can be used to identify suitable environmental conditions across great geographic extents. Carefully implemented SDM studies may offer important guidance for rewilding projects in terms of species and area selection. However, only a few studies have taken this approach to date [85–87]. A single SDM study has evaluated climate suitability in Australia to assess the potential distribution and impact of feral horses in Queensland, Australia, but from an invasive species perspective [88].

With this study, we provide an assessment of the distribution of suitable climate and habitat for wild-living *E*. *ferus* worldwide based on SDM analyses of a global dataset of the distribution of current wild-living populations. We use the term wild-living to refer to feral and semi-feral horse populations as well as reintroduced wild-living populations of *E*. *f*. *przewalskii*. Feral horses are escaped horses with no owners roaming free in natural areas. Semi-feral horses are wild-living horses with owners, who care for them if necessary. The main research questions are: 1. What are the climatic factors characterizing wild-living *E*. *ferus* habitats? 2. What is the geographical distribution of suitable climate and habitat for *E*. *ferus* worldwide and within its former range? 3. Which areas are suitable for rewilding with *E*. *ferus* when also considering the biogeography and ecology of other equids? and 4. Finally, how much suitable habitat for *E*. *ferus* occurs within five major rewilding areas in Europe (several with planned *E*. *ferus* reintroductions, one with a long-established feral horse population, and one with two recently established populations)?

## Methods

### Occurrence data on feral and semi-feral *E*. *ferus*

Data on the distribution of feral *E*. *ferus* worldwide were gathered from government agency and NGO web sites, scientific and grey literature, and personal communication with researchers and others working with feral or semi-feral *E*. *ferus* (see S1 Table for details). Only information on the distribution of feral and semi-feral *E*. *ferus* that have been present during all or part of the period from the year 2000 forward was used. In addition to geographic information, we also obtained information on management methods, e.g., provisioning of supplementary food and water, and fencing.

**Fig 2:**
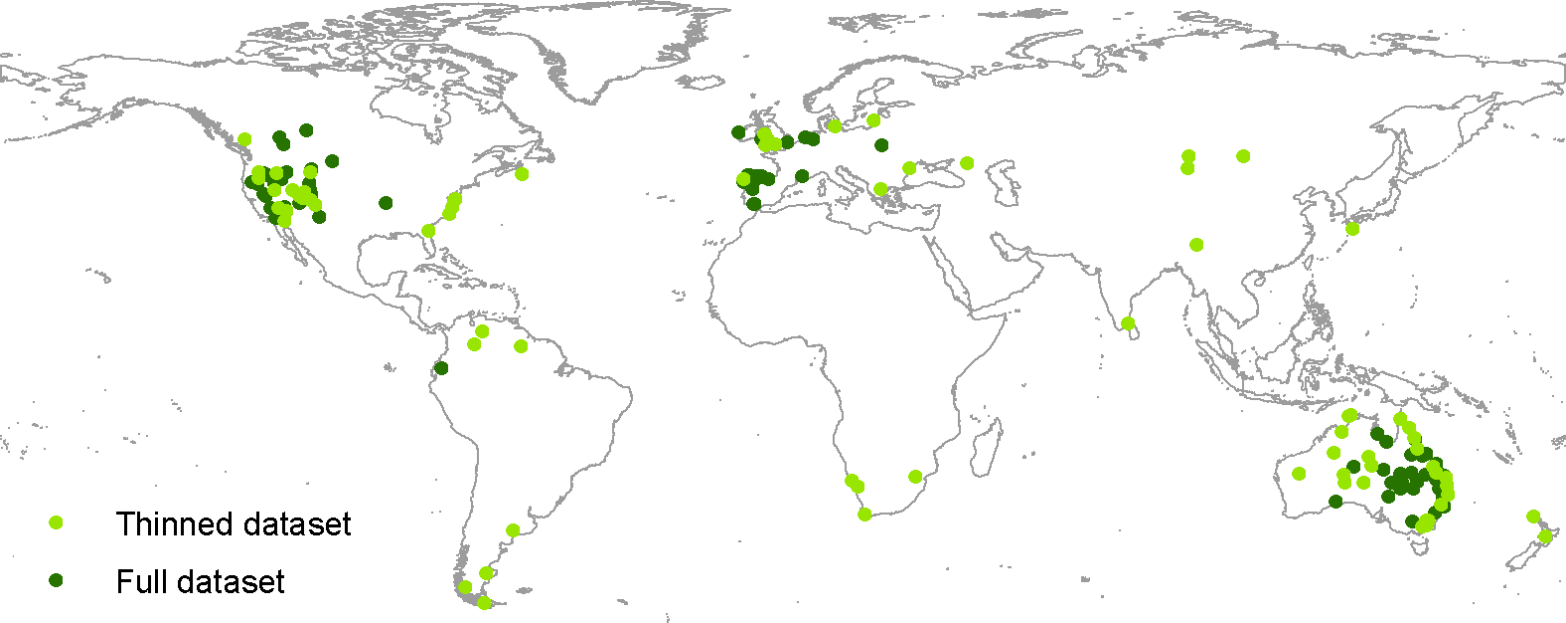
Current distribution of wild-living horses (*E*. *ferus*). The dots indicate the 186 populations of recent wild-living *E*. *ferus* identified in this study. Light green, included by the OccurrenceThinner procedure (n = 76); and dark green, excluded (n = 110) (see the text for further details).

Populations were considered semi-feral rather than feral if one of the following criteria were met: the area was fenced; there were supplementary water and/or occasionally food available to them; or they were checked or treated by veterinarians (deworming) or blacksmiths (hoof trimming). To qualify as semi-feral, a population should still be kept outside all year. The majority of the semi-feral populations were breeding stock of purebred *E*. *ferus* located in Europe. Semi-feral populations restricted to areas less than 171 ha were excluded from the analysis. This is the minimum area known to be occupied by a fully feral population [restricted to an entire island] persisting for at least 40 years, namely on Tærø, Denmark [89] (personal communication with the game keeper at Tærø). Three populations of Asiatic wild horse (*E*. *f*. *przewalskii*) were included in the dataset. The current distribution of wild-living *E*. *ferus* is shown in Fig 2.

Shape files of areas inhabited by wild-living *E*. *ferus* (feral horse range areas) were obtained from the literature or informant sources if possible. If such were not available, the areas were digitized from various types of maps, such as tourist brochures, national parks boundaries in the Bing World map (www.Bing.com) and/or written descriptions of the extent of the feral horse range area. All shape files were created in or projected into the geographic coordinate system WGS1984. Shape files of populations managed by the Bureau of Land Management (BLM) in the western United States of America (US) show administrative unfenced boundaries rather than actual home ranges of feral *E*. *ferus* populations. Horses migrate among these areas, so all areas within a 16 km distance of each other were merged (16 km is the average distance travelled a day by Australian feral *E*. *ferus* [90]). For the rest of the dataset, areas sharing administrative boarders were merged. Environmental data were extracted for the centroids of the shape files to ensure that each feral horse range area received the same weight in the subsequent analyses. For the feral *E*. *ferus* population in Kaapsehoop, Mapumalanga province, South Africa, the only information available was the approximate area occupied by feral *E*. *ferus* and that they lived literally next to the village of Kaapsehoop. For this population, we extracted the climatic data at the coordinates for the village of Kaapsehoop.

### Modelling

In summary (see detailed description below), we used SDM to identify areas with a climate suitable for wild-living *E*. *ferus* worldwide. To do this, we used Maximum Entropy (MAXENT) modelling [91] as we considered our data to be presence-only data. The MAXENT models were built with climatic variables, and their predictive performance was evaluated using the True Skill Statistic (TSS). We also generated a simple rectilinear Bioclim-type climatic envelope model (CEM) [92] to verify the robustness of the MAXENT results as different modelling techniques may provide different predictions [93]. We used land cover to filter the climatic predictions, i.e., to delimit the areas with suitable land cover within the extent of the final MAXENT models. Finally, we assessed the suitability of five large areas proposed for rewilding in Europe based on the final MAXENT models and land cover.

To model areas with habitat suitable for wild-living *E*. *ferus*, we used MAXENT version 3.3.3 (http://www.cs.princeton.edu/~schapire/maxent/). MAXENT is one of the best methods for modelling species distribution with presence-only data [91, 94]. We used default values for the convergence threshold (10^-5^) and the logistics output format, which ranges from 0–1 and can be interpreted as a relative probability of presence [95]. Maximum iterations were set to 5000. Differences in management and available information on wild-living *E*. *ferus* populations worldwide might lead to geographic sampling biases. Further, the areas occupied by many feral horse populations today might more reflect available habitat and human persecution than optimal habitat, cf. the refugee species concept [96, 97]. To minimize these effects, we removed some of the populations in the areas with the highest concentration of wild-living *E*. *ferus* populations using OccurrenceThinner version 1.04 [98], which reduces geographical bias in datasets by a probability-based procedure. The probability that an occurrence record is removed is proportional to the density of occurrences in its neighbourhood as defined by a kernel density grid. OccurrenceThinner was run with a lower threshold of 0 and an upper threshold of 1. Ten replicate runs were performed, yielding 10 thinned datasets. These datasets were then compared. Those lacking populations in areas with a low density of wild-living *E*. *ferus* populations were excluded, and the dataset with the most geographically even occurrence densities was retained for the analysis. The thinned dataset consists of 76 occurrences vs. 186 in the full dataset. For a comparison between the thinned and full datasets, see Fig 2, S1 and S2 Figs.

### Predictor variables

Initially, we considered eight environmental variables (Table 1), representing climatic factors that plausibly might affect the distribution of *E*. *ferus*. The climatic variables were extracted from the WorldClim database at a 10-km resolution for the period 1950–2000 (http://www.worldclim.org/current) [99]. Maximum and minimum values for the initial environmental variables were the same for the full and thinned datasets (see S2 Table).

**Table 1:** Initial set of environmental variables with range values at 10-km resolution for the thinned dataset used for the analysis.

Variables	Code	Values
Mean temperature of the wettest quarter (°C)	MTWeQ	-3.0–31.5
Mean temperature of the driest quarter (°C)	MTDQ	-18.2–29.0
Mean temperature of the warmest quarter (°C)	MTWQ	6.6–32.1
**Mean temperature of the coldest quarter (°C)**	**MTCQ**	**-22.9–26.5**
Total precipitation of the wettest quarter (mm)	PWeQ	26.0–1541.0
**Total precipitation of the driest quarter (mm)**	**PDQ**	**2.0–300.0**
Total precipitation in the warmest quarter (mm)	PWQ	13.0–1380.0
**Total precipitation in the coldest quarter (mm)**	**PCQ**	**3.0–897.0**

**Fig 3:**
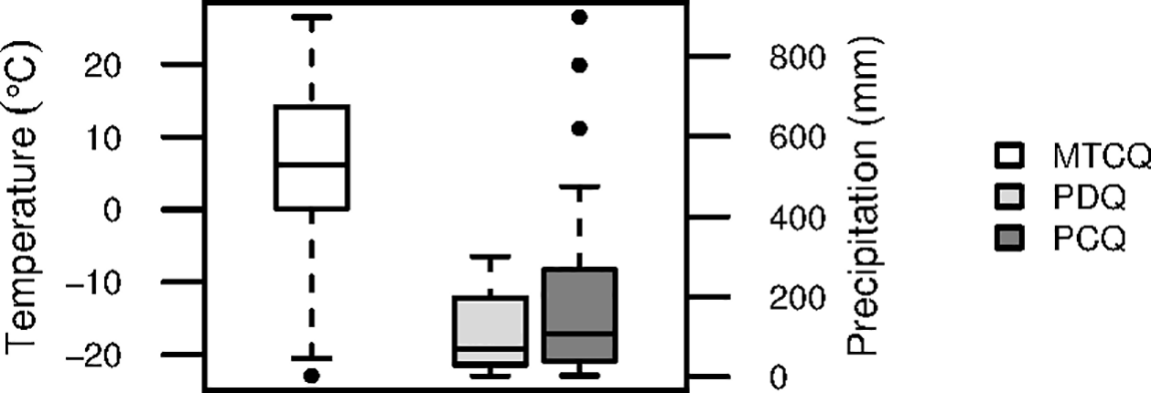
Analysis of feral horse occurrences using species distribution modelling. MAXENT species distribution modelling of the selected wild-living horse (*E*. *ferus*) localities (n = 76) worldwide using climatic predictor variables. (**A**) Test gain jackknife evaluation of the relative importance of all variables considered; (**B**) Test gain jackknife evaluation of the relative importance of the three selected variables; and (**C**) Estimated response curves (showing the probability of presence using the logistic output from MAXENT). For acronyms, see Table 1.

To assess variable importance and to develop a predictive distribution model, we fitted and evaluated MAXENT models including all predictor variables. We then simplified the models by removing the variables with little or no predictive power (little or no contribution to the model according to the jackknife evaluation of test gain (Fig 3A)). As correlations among variables might jeopardize the interpretability of the results, we used a pairwise Pearson’s correlation test to examine the correlation between variables (S3 Table). For predictor variables with Pearson’s r < 0.65, we kept the variable with the strongest biological interpretability and excluded the others. Three variables were thereby selected for the final modelling: mean temperature in the coldest quarter (MTCQ), precipitation in the coldest quarter (PCQ), and precipitation in the driest quarter (PDQ). MTCQ had Pearson’s r values of 0.205 and -0.118 with PCQ and PDQ, respectively, whereas PCQ and PDQ together had r = 0.583. Mean temperature of the driest quarter (MTDQ) had a higher test gain than did MTCQ, but the two variables were correlated (r = 0.817). MTCQ did, however, have a stronger biological interpretability as MTCQ influences the amount of forage, capturing temperatures too low for plant growth and snow cover (in combination with PCQ in areas with MTCQ ≤0°C). MTCQ also provided better prediction of the Holocene distribution of *E*. *ferus* than MTDQ (S3 Fig).

Predictions of suitable habitat have been shown to be more robust using ensemble approaches [100]. We therefore generated an ensemble prediction with the final MAXENT models (TSS ≥ 0.410, see Model validation for further information). To study the effects of different suitable-unsuitable thresholds for the MAXENT probabilities, we also generated ensemble predictions with three different thresholds on the final models: minimum training presence (classifying all localities with climatic scores greater than the minimum values for the feral horse localities as suitable); 10% training presence (to account for possible extremes caused by uncertainties in the data collection) and equal sensitivity and specificity (where the likelihood of a false positive is set to balance the likelihood of a false negative [101]). Different model techniques can yield different results in species distribution modelling [93]. The CEM was fit with the variables used in the final MAXENT (TSS ≥ 0.410). The bioclimatic envelope model was built by finding the maximum and minimum values of each of the variables for the occurrences in the dataset. We then identified areas worldwide that fell into the range of each variable. Finally, we generated a combined CEM map of these predictions, indicating where all of the variable ranges overlap. All GIS operations were performed in ArcGIS 10.1/10.2 (ESRI, Redlands, CA). All background maps of the world are based on the world boundaries map from OpenStreetMap (www.openstreetmap.org).

### Model validation

We used the true skills statistic (TSS) to evaluate the accuracy of the models. TSS is a variant of the Kappa statistic but, in contrast, is insensitive to prevalence [102]. The identification of suitable rewilding sites requires maps of suitable vs. unsuitable conditions. To assess the predictive power, we split the data into 50% training data and 50% test data. This partitioning has been demonstrated to be the most robust [103]. When modelling species distributions, one needs to specify a threshold at which the habitat is considered suitable to evaluate model performance [102]. Different thresholds can have a great effect on which areas are mapped as presences and which as absences [104]. We used a threshold of a minimum 10% training presence for the TSS. Landis & Koch [105] suggest the following benchmarks for kappa statistics: 0.000–0.200: slight, 0.200–0.400: fair, 0.410–0.600: moderate, 0.610–0.800: substantial, and 0.810–1.000: almost perfect. We used models with TSS ≥ 0.410 to model areas suitable for the rewilding of wild-living *E*. *ferus*. AUC is often used to assess the predictive power of SDMs, but accurate calculation of AUC values requires both presence and absence data [91, 102]. It does, however, still give a sense of the predictive power [91], with AUC values of 0.5–0.7 having low accuracy, 0.7–0.9 having useful applications and > 0.9 high accuracy [106].

### Suitable habitat

To account for the effect of land cover restrictions on habitat availability, we used the GlobCover land cover dataset [107]. At the global and regional scales, we used an aggregated dataset at a 10-km resolution. The 10-km resolution was obtained by first aggregating the original GlobCover dataset from a 300-m resolution into a 9-km resolution. This was completed by aggregating the original 300-m by 300-m cells into 9-km by 9-km cells, using the Aggregate tool in ArcGIS 10.1 set to “Majority” (the land use category in the 10-km cell is the most abundant category in the original 300-m cells aggregated into the 10-km cell). We then used resample (nearest neighbour) to generate the final 10-km resolution land cover map. For Rewilding Europe areas (see below), we used the original 300-m resolution GlobCover land use dataset. The land use categories were chosen based on the habitat use of current wild-living *E*. *ferus* (see Introduction). We mapped two kinds of habitat: primary habitat and secondary habitat. Primary habitats are the types of habitat that are ideal for wild-living *E*. *ferus* and necessary to survive and reproduce. These are open habitats such as grasslands, savannas and scrub [41, 49, 59–61, 63, 64, 66–69, 71, 73, 77, 108]. Secondary habitats are less ideal habitats that wild-living *E*. *ferus* may also utilize or which might connect patches of primary habitat. Secondary habitat is comprised of open to closed forests and woodlands [66, 68, 71, 77]. We mapped the distribution of primary and secondary habitats within the estimated suitable climate range according to MAXENT models with a TSS > 0.410. The categories are shown in S4 Table. The raster with the extent of the two models was resampled to have same resolution as the GlobCover data (10-km for global and regional scales and 300-m for the Rewilding Europe areas).

### Application to assessments of habitat suitability within local rewilding areas

At the landscape scale, we assessed the availability of suitable habitat for wild-living *E*. *ferus* in five areas in Europe: Western Iberia in Spain (1.75 m ha) and Portugal; the Eastern Carpathians in Poland, Slovakia and Ukraine (226,000 ha); the Southern Carpathians in Romania (208,000 ha); the Danube Delta in Romania and Ukraine (633,000 ha) and Velebit in Croatia (249,000 ha). These areas are part of the Rewilding Europe initiative, an initiative aimed at creating large wilderness areas in Europe (www.rewildingeurope.com). In these areas, Rewilding Europe wishes to re-establish naturally grazed ecosystems with large herbivores such as *E*. *ferus*. Two of these areas, Western Iberia and the Danube Delta, already contain populations of feral *E*. *ferus* [54]. *Equus ferus* has been observed to avoid areas with a slope of more than 30° [109]. To account for this issue, we mapped areas with slopes of 30° or more in the five rewilding areas as unsuitable. The area of primary habitat in each rewilding area was estimated by calculating the percentage of the area covered by primary habitat in ArcGIS 10.2 (ESRI, Redlands, CA).

## Results

The MAXENT species distribution modelling indicated that the occurrence of wild-living *E*. *ferus* is related to MTCQ, PDQ and PCQ, with MTCQ as the strongest predictor, followed by PDQ (Fig 3C). The model validation showed the two models to have TSS ≥ 0.410: A model comprised of all three predictor variables and a model including MTCQ and PDQ (Table 2). These are the two final models.

**Table 2:** The five MAXENT models for wild-living *E*. *ferus*.

Model	MTCQ	PCQ	PDQ	AUC test	TSS	Threshold
**1**	X	X		0.794	0.345	0.225
**2**	X		X	0.808	**0.463**	0.311
**3**	X	X	X	0.814	**0.503**	0.380
**4**	X			0.779	0.349	0.345
**Full** *				0.866	**0.508**	0.286

* Built from the eight initial variables: MTWeQ, MTDQ, MTWQ, MTCQ, PWeQ, PDQ, PWQ and PCQ. For acronyms see Table 1.

**Fig 4:**
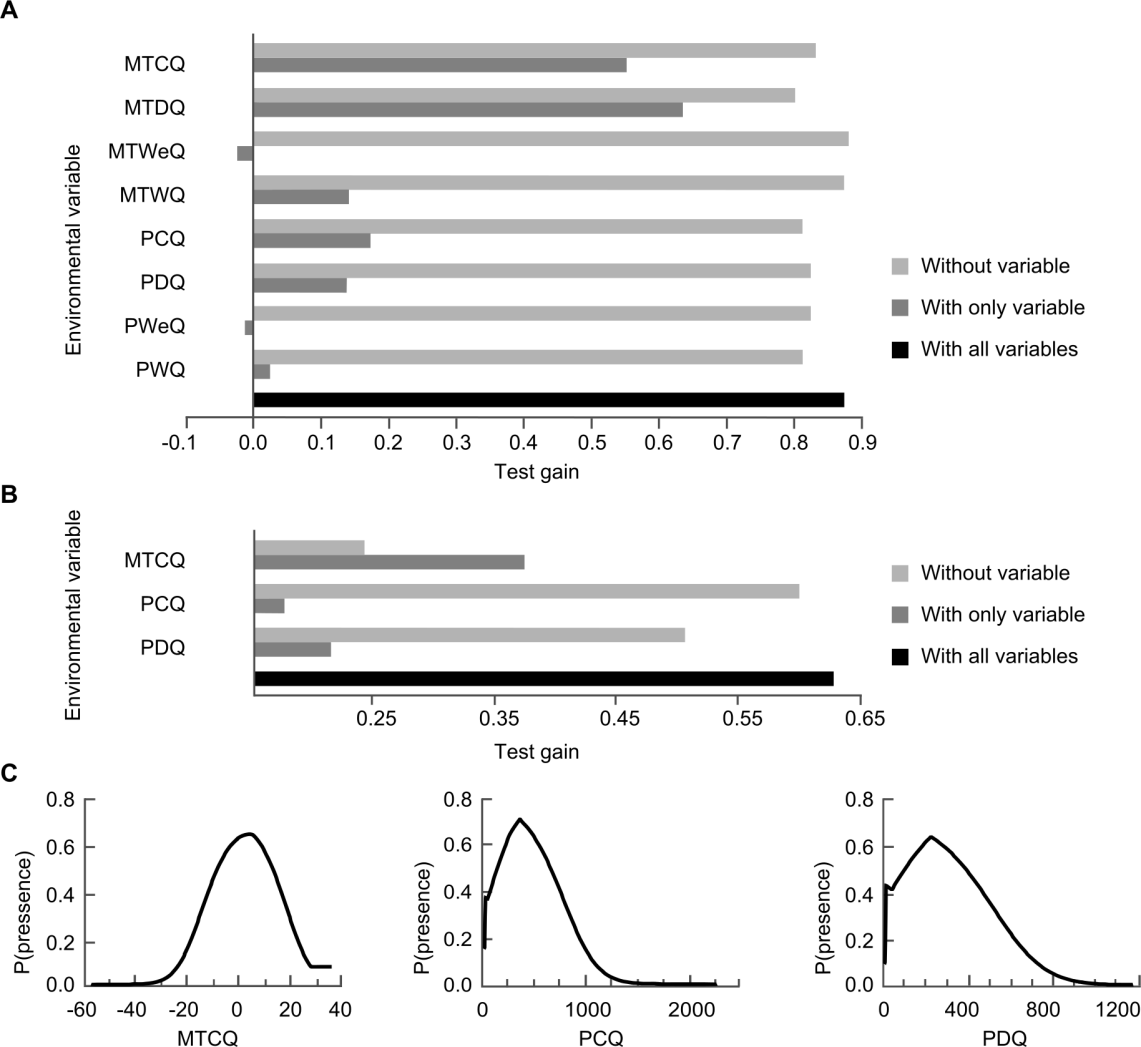
Boxplots of the three focal climatic variables for wild-living horse (*E*. *ferus*) localities (n = 76) worldwide. Mean temperature in the coldest quarter (MTCQ) (white), precipitation in the driest quarter (PDQ) (light grey) and precipitation in coldest quarter (PCQ) (dark grey). Whiskers mark the 1^st^ and 3^rd^ quartiles, and the line indicates the median. Dots are outliers.

**Fig 5:**
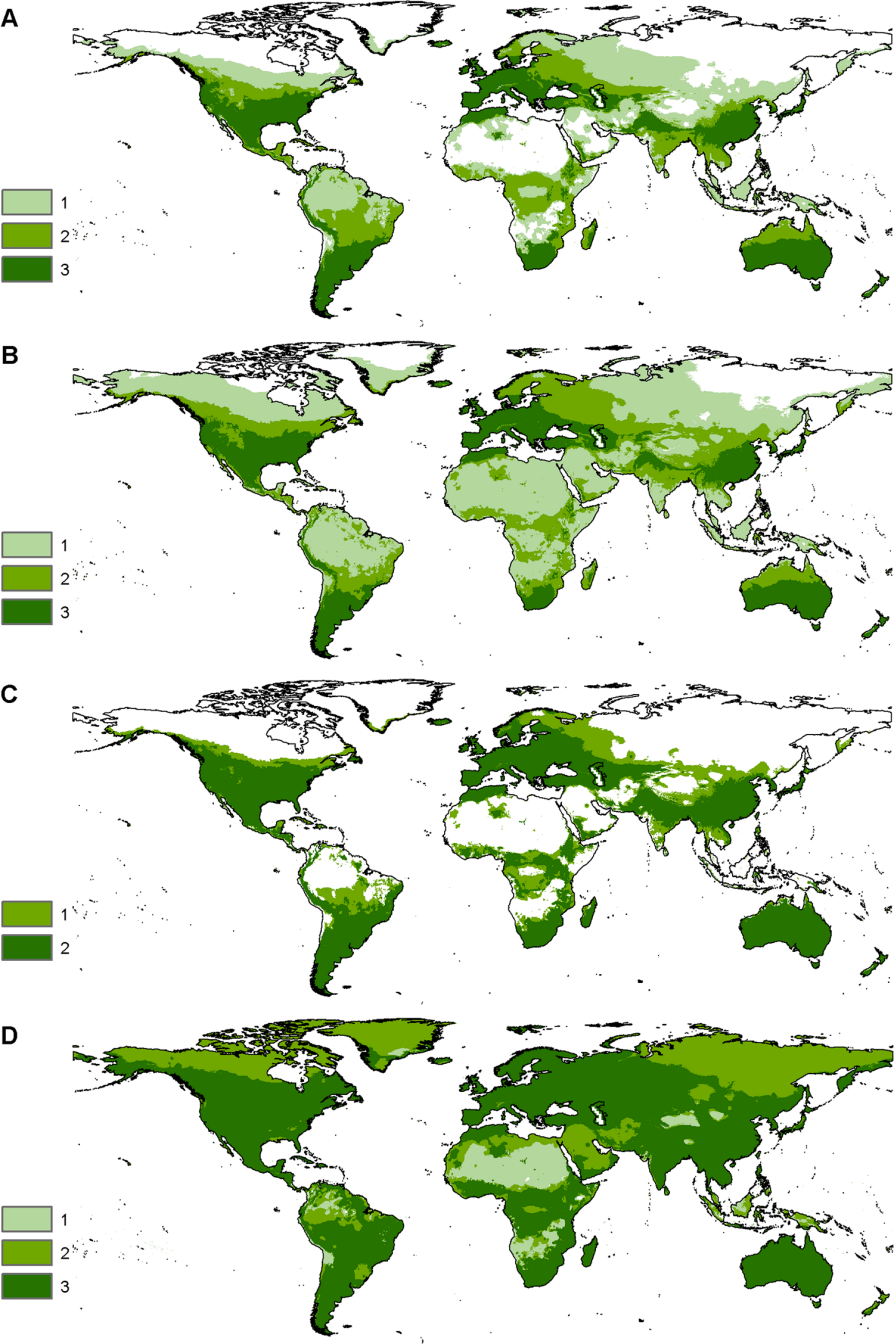
Areas with suitable climate for wild-living horse populations worldwide. MAXENT predictions of areas with a suitable climate at a 10-km resolution for models based on (**A**) mean temperature in the coldest quarter (MTCQ) and precipitation in the driest quarter (PDQ), and (**B**) MTCQ, PDQ and precipitation in the coldest quarter (PCQ), using the 76 selected wild-living horse (*E*. *ferus*) localities. The maps show overlap in the predictions of suitable climates at three presence-absence thresholds: minimum training presence, minimum 10% training presence and equal sensitivity and specificity. The colours indicate the number of threshold criteria predicting a suitable climate for each grid cell ranging from 1–3. (**C**) Ensemble map showing the overlap of the predicted suitable climates for the two final models for each grid cell, based on the 10% training presence threshold (Table 2). (**D**) Predicted suitable climate from the CEM. Colours indicate the number of overlapping climatic variable ranges for each grid cell, ranging from 1–3.

Even considering these three variables, wild-living *E*. *ferus* clearly occur under a wide variety of climatic conditions (Fig 4). The MAXENT models accordingly also indicated suitable climate for wild-living *E*. *ferus* in large areas of the world (Fig 5A–5C). In the Americas, these areas included most of the United States and the southern parts of Canada as well as much of Central and South America, excluding the Amazon. In Eurasia, suitable climates occur in most of Europe and parts of the Middle East, India and Asia, excluding Southeast Asia, western China and the southern part of Siberia. The northern, middle and southern parts of Africa are suitable as well, excluding the Sahara, parts of eastern and central Africa and much of the central African rainforest. Finally, most of Australia and New Zealand are suitable (Fig 5). The CEM modelling approach resulted in a prediction of similar areas with suitable climate as the MAXENT models, albeit including more of Siberia (Fig 5D).

**Fig 6:**
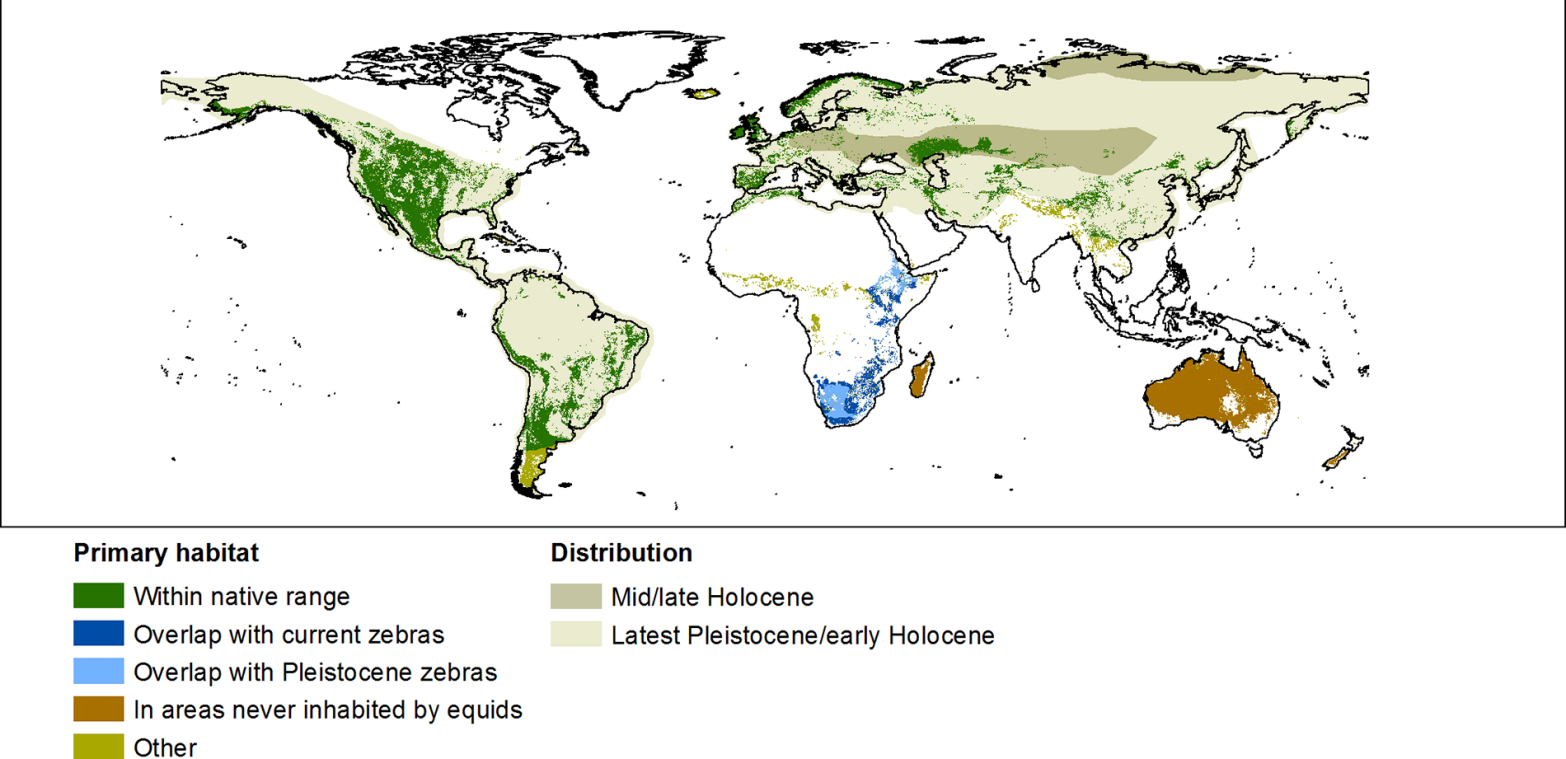
Potential habitat (suitable land cover) for feral horses (*E*. *ferus*). The distribution of primary habitat within and outside the native range of *E*. *ferus* at a 10-km resolution within the extent of the final MAXENT models, showing the primary habitat outside of the native range of *E ferus* overlapping with the current [41] and Pleistocene distributions of zebras [41, 157] in Africa; in areas never inhabited by equids (Australia, New Zealand and Madagascar); outside the native range of *E*. *ferus* in areas previously inhabited by equids and outside the current and Pleistocene distribution of zebras (Other). The distribution of *E*. *ferus* during the Pleistocene (ca 1.1 MA to 15,000 BC); the Pleistocene/Holocene transition (15.000 to 3500 BC) (modified from [25]) and mid/late Holocene (3500 BC-present) are also shown. For maps of past distributions of *E*. *ferus*, the distribution of primary habitat and the distribution of other extant equids only, see S4 and S5 Figs.

Using land cover to identify primary *E*. *ferus* habitat within the areas of suitable climate predicted by the two best MAXENT models revealed suitable areas in the United States, central and southern South America, Europe, south-western Russia and Kazakhstan, south-western China, Southern Africa and Madagascar, Australia and New Zealand (Fig 6).

**Fig 7:**
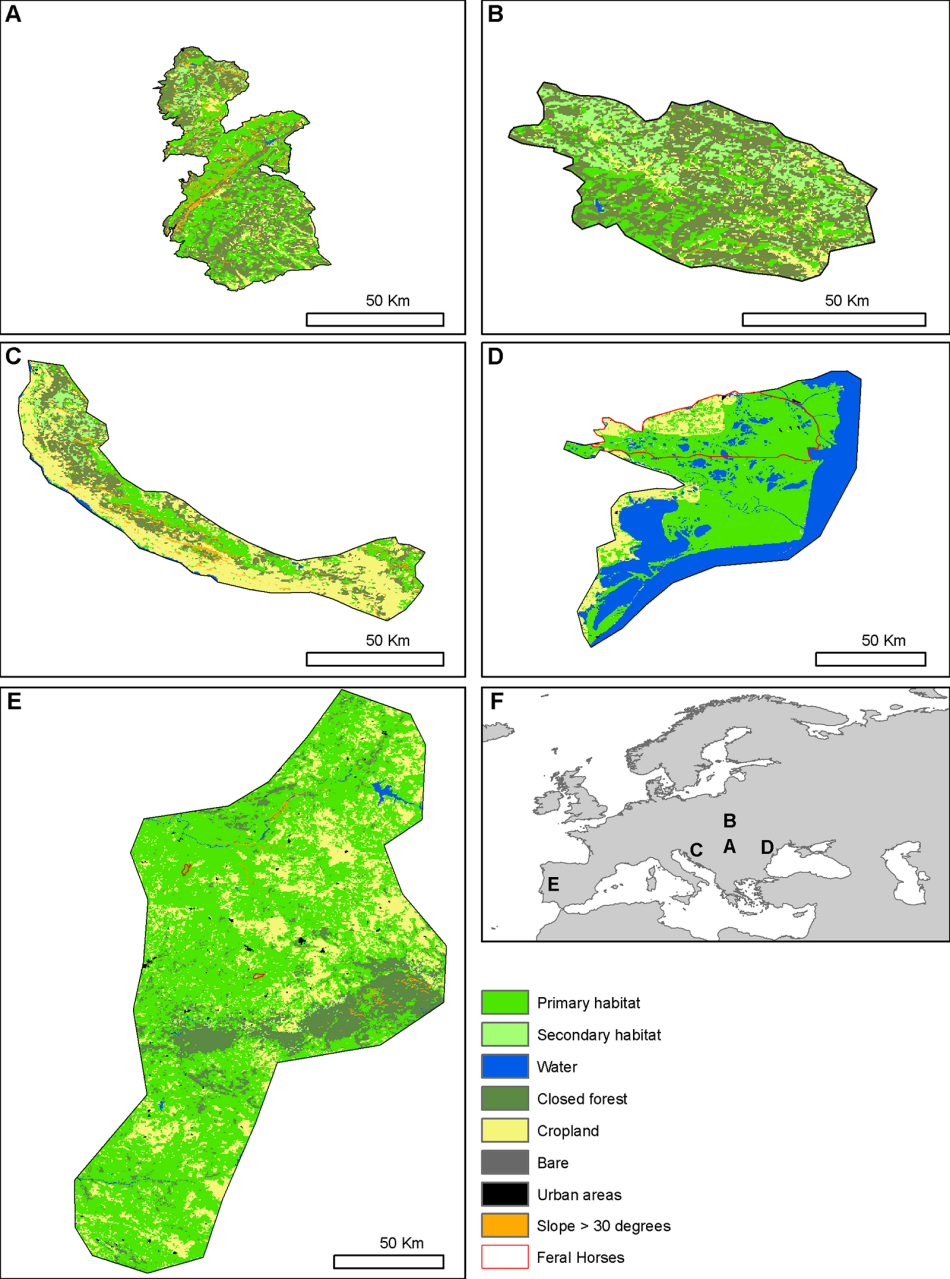
Suitable habitat for wild-living horses (*E*. *ferus*) in five major rewilding areas in Europe. (**A**) Southern Carpathians. (**B**) Eastern Carpathians. (**C**) Velebit. (**D**) Danube Delta. (**E**) Western Iberia. (**F**) Locations of the five areas in Europe (**A**-**E**). Colours depict primary habitat (highly suitable land cover), secondary habitat (non-essential land cover occasionally utilised), unsuitable habitat (slopes of 30° or more, closed forest, urban areas, bare areas and cropland) and water.

All five major European rewilding areas are characterized by a suitable climate for wild-living *E*. *ferus* according to the two best MAXENT models and contain abundant primary and secondary habitat (Fig 7). Notably, primary habitat covers 19–66% of these areas, corresponding to 42,000–1,150,000 ha (Table 3).

**Table 3:** Suitable habitat availability for wild-living *E*. *ferus* within five major rewilding areas in Europe.

Region	Suitable habitat (ha)	Primary habitat (ha)
Southern Carpathians	226,000	86,000
Eastern Carpathians	208,000	42,000
Velebit	249,000	47,000
Danube Delta	633,000	329,000
Western Iberia	1,750,000	1,156,000

## Discussion

### Climatic determinants of the distribution of wild-living *E*. *ferus*

The current distribution of wild-living *E*. *ferus* worldwide was analysed as a function of climate. The two best performing models included combinations of Mean Temperature of the Coldest Quarter (MTCQ), Precipitation in the Driest Quarter (PDQ) and Precipitation in the Coldest Quarter (PCQ). These models were able to delimit the current distribution of wild-living *E*. *ferus* quite accurately on a coarse global scale, suggesting that climate is an important factor for the worldwide distribution of wild-living *E*. *ferus*. At the global and continental scales, climatic factors are often strong predictors of species distributions, whereas other factors such as biotic interactions, land cover and soil type are often better at explaining species distributions at small scales [110, 111]. Still, biotic interactions may also shape distributions at large scales [111]. Nevertheless, it is clear that wild-living horse populations occur under a very broad range of climatic conditions.

Mean Temperature of the Coldest Quarter was most important among the three climatic predictor variables (Fig 3B) and limited horse distributions to the north. Horses are rather resilient to cold temperatures, and the effects of MTCQ on *E*. *ferus* are most likely through food availability [67]. In this analysis, we used both feral and semi-feral populations of *E*. *ferus*. Semi-feral populations sometimes are given additional fodder during harsh winters, which could bias the models towards climates that are too harsh for horses to survive on their own. Still, all populations supplied with fodder during winters are within the Holocene distribution of *E*. *ferus*; thus, the species has been able to maintain itself in the wild in these areas under climates similar to the present. Horses are kept on ranges year-round in Yakutia, Russia, where MTCQ can be as low as -44°C [99, 112]. Horses have been observed to dig through up to 60 cm of snow, but a deeper snow cover for a prolonged period may cause starvation [67, 108, 113]. Low MTCQ (from approximately 0°C and lower) results in a higher risk of precipitation falling in the form of snow, and PCQ determines the depth of snow cover in regions with daily temperatures mostly less than 0°C during the coldest quarter. Looking at feral populations with MTCQ less than 0°C, there seems to be a tendency for lower PCQ with lower MTCQ, i.e., that horses in cold areas occur where there is relatively little snowfall.

Precipitation in the Driest Quarter probably acts on horse distributions via effects on vegetation and the amount of water available for drinking during dry seasons. Unless they are very cold, areas without any dry period will typically harbour forests [114], and dense forests are not a suitable habitat for wild-living horses (but see later discussion). Horses are not as drought-adapted as certain other equids. In contrast to *E*. *africanus* (donkeys and wild asses), the kidneys of *E*. *ferus* contain few loops of Henle and thus cannot produce strongly concentrated urine [25]. However, Hampson et *al*. found that wild-living *E*. *ferus* in semi-arid areas travel up to 55 km or 12 hours between water source and grazing [90], allowing them to inhabit arid areas as long as there are water sources nearby. Many of the populations of wild-living *E*. *ferus* inhabiting desert environments have access to man-made water reservoirs, which may affect the estimated relationship to PDQ. Water is not transported into these areas, but rainfall accumulates in these reservoirs, allowing access to drinking water during periods of drought.

### Geographic distribution of suitable climate and habitat for wild-living *E*. *ferus*

The geographical extent of presence/absence maps of suitable conditions estimated by species distribution modelling will depend on the threshold criteria selected [104], and different criteria may result in large differences in predicted presence/absence [103]. In this study, we modelled the distribution of suitable climatic conditions for wild-living *E*. *ferus* with three different threshold criteria for the two best MAXENT models (Fig 5): minimum training presence, 10% training presence, and equal sensitivity and specificity. The resulting presence/absence maps revealed large differences in the extent of suitable climate, with the minimum training presence threshold being the least conservative and the equal sensitivity and specificity threshold being most conservative. The minimum training presence threshold estimates suitable climate in areas outside the Pleistocene distribution of *E*. *ferus* (the northern part of Canada, Greenland, Middle East and Asia), in areas with tropical rainforests in South America, Central Africa and Asia, and in deserts and, thus, is probably overpredicting. This threshold does, however, capture much more of the Pleistocene and Holocene distribution in the Eurasian steppes than the other thresholds. The 10% training presence threshold captures the current distribution of *E*. *ferus* fairly well, notably in North America, western and southern Eurasia, southern South America, Australia and sub-Saharan Africa, although it fails to predict the three reintroduced populations of Asiatic wild horse (see discussion above). The projection of suitable climate is in good agreement with the Pleistocene and Holocene native distribution of *E*. *ferus*, with some notable exceptions. The former range in the central and north-eastern parts of the Eurasian continent is not predicted to be suitable, and certain regions beyond the former native distribution have large suitable areas, notably Australia, New Zealand, and Sub-Saharan Africa (Fig 5C). The equal sensitivity and specificity threshold is obviously too conservative as it fails to capture large areas with current populations of wild-living *E*. *ferus* in North America, Eurasia and Australia. This illustrates that presence-absence maps of species distributions should be created with careful consideration of the selection of threshold criteria, notably if the resulting maps are to be used to guide conservation management.

Looking at the overlap of the rectilinear CEMs between MTCQ, PDQ and PCQ (Fig 5D), this method seems to predict suitable climate in areas not currently inhabited by wild-living *E*. *ferus*, but within the latest Pleistocene and Holocene distributions of the species. This method only considers maximum and minimum values of the climatic variables and is thus less affected by bias in sampling effort and information availability in general. It does, however, fail to exclude the dense Amazonian and Central African rainforests, which are clearly not suitable habitats for *E*. *ferus*.

Concerning central and north-eastern Eurasia, the three reintroduced populations of *E*. *f*. *przewalskii* existing there today fall outside the areas estimated to be climatically suitable, indicating that the modelling is too conservative here, which is also suggested by the fact that this region encompasses major late Holocene strongholds for wild *E*. *ferus* (Fig 6A). The limited estimated suitable climate in Siberia is most likely caused by the many wild-living *E*. *ferus* populations in western North America, Europe and Australia. These areas are either temperate and moist (Europe) or temperate to tropical and dry (western North America and Australia), whereas Siberia is boreal to arctic. This bias stems from differences in available information on wild-living *E*. *ferus* in different regions of the World and differences in where socioeconomics and culture has allowed wild-living *E*. *ferus* populations to establish. Notably, human activities probably exclude *E*. *ferus* from areas and habitats where it was formerly abundant, e.g., productive steppe areas that are now under intense agriculture. Hence, wild-living *E*. *ferus* may constitute a refugee species, with a distribution that human activities skew towards formerly marginal environmental conditions [17, 115]. In such cases, species distribution modelling may fail to predict areas that in reality have high suitability [91, 97, 116–123]. Furthermore, in some regions such as much of central and north-eastern Eurasia, feral *E*. *ferus* are valuable assets for local populations relying on herding, and feral individuals will often be captured and domesticated. The Asiatic wild horse (*E*. *f*. *przewalskii*) became extinct in the wild in the 1960s in its last area of occurrence, the Mongolian part of the Gobi desert. It has since been reintroduced to this area [55]. It is debated whether this area is actually optimal habitat or if it was simply the last refuge [115]. Studies of stable isotopes and morphology in Pleistocene *E*. *ferus* suggests that the species was found primarily in open habitats as a grazer [38, 124–128], but a few fossils suggest more forested habitats and a mixed grazer-browser diet [124, 129–131]. *E*. *ferus* has been found in both glacial and temperate periods in habitats varying from glacial open steppe to interglacial forest landscapes [124–126, 129–134]. However, Pleistocene interglacial forest landscapes likely often harboured extensive open and semi-open areas due to the activities of the rich fauna of large herbivores [88]. Today, wild-living populations of *E*. *ferus* are found in various open habitats, primarily in grasslands and scrub, but also in salt marshes, wetlands, deserts, heathlands, open woodlands and mosaic forest-open vegetation [41, 49, 53, 59, 60, 62–79, 135]. Neighbouring, more densely forested areas are often used as shelter in winter storms [66, 67, 71, 77]. In accordance with this, Sommer *et al*. [38] suggest that fluctuations of populations of *E*. *ferus* in Holocene Europe can be explained as a response to varying vegetation openness, with Holocene landscapes likely more densely wooded than during the Pleistocene interglacials due to a reduced large herbivore fauna [88]. The morphology and paleoecology of *E*. *ferus* suggest that it also occurred in cooler and more mesic habitats than feral populations today [124, 125, 130, 131, 136]. In summary, the current realized niche of wild-living *E*. *ferus* is most likely only a subset of the potential niche of the species, likely being biased towards warm and dry areas due to human activities.

Climate is not the only variable affecting the suitability of an area for wild-living horses. Habitat, home range size, intra- and interspecific relations and conflicts with humans are also important when assessing suitable habitat for rewilding or reintroduction. These factors are likely to have a dominant role at landscape scale [110]. Species depend on the availability of food, water and, sometimes, specific habitats. Kuemmerle *et al*. found that the best prediction of suitable habitat for European bison (*Bison bonasus*) within its historical range across Europe was modelled on land cover, anthropological effects and physical characteristics of the area [85]. In this study, we used information on preferred habitats from the literature to estimate the primary habitats as our data contained little information on habitat use in most of the feral horse range areas. In the mapping process, we excluded all areas at a 10-km resolution that included suitable land cover types for wild-living *E*. *ferus* together with anthropogenic land cover types such as cropland to not include in our estimate areas that are clearly suitable for rewilding with horses where there is risk of conflicts with humans. Areas with suitable climate and habitat are primarily found in the US, central and southern South America, Europe, south-western Russia and Kazakhstan, south-western China, sub-Saharan Africa and Madagascar, Australia and New Zealand (Fig 6). However, only considering regions with large areas of suitable habitat provides a too conservative picture as many small-scale rewilding projects with horses occur in densely populated parts of Europe, such as the Netherlands [137].

### Zoogeographic perspectives on the potential for rewilding with wild-living *E*. *ferus*

Whether a reintroduction is desirable depends on several factors, including little competition between the reintroduced species and native species with similar ecologies. In the case of rewilding with *E*. *ferus*, it is therefore pertinent to consider the former native distribution of *E*. *ferus*, the present and recent past distribution of other equid species and the overall global distribution of equids in general. Within the former Pleistocene range of *E*. *ferus*, there is little reason to suspect general negative effects on other native species as the vast majority will have coexisted with *E*. *ferus* for 10^5^-10^6^ years in the past and with other grazing equids for even longer [35, 36]. Concerning other equids, *E*. *ferus* previously coexisted with a number of extinct and extant equids (Fig 6), notably stilt-legged horse (*E*. *semiplicatus* and allied taxa) in North America, *Hippidion* spp. in South America, and European wild ass (*E*. *hydruntinus*) [22, 34, 46, 127, 134, 138], as well as extant Asiatic wild asses (*E*. *hemionus* and *E*. *kiang*) in Asia [41] and African wild ass (*E*. *africanus*) in northern Africa [139]. Feral *E*. *ferus* currently share habitat with feral asses (*E*. *africanus*) in Western North America and Australia. The two species are quite different in ecology and behaviour and do not generally compete with one another [65]. Both species are found in open habitats, but *E*. *africanus* is better adapted to hot arid climates [39, 41, 140]. *Equus ferus* and *E*. *africanus* also differ in behaviour, with *E*. *ferus* forming stable bands and not maintaining territories [67, 140, 141], whereas *E*. *africanus* is territorial and does not form stable bands [140]. These behavioural traits in *E*. *africanus* are thought to be an adaption to low availability and quality of forage in arid environments [41]. Similarly, *E*. *f*. *przewalskii* occurs within the known distribution of *E*. *hemionus* in Mongolia and northern China. *Equus hemionus* is a mixed-feeder and is able to live on woody plants but feeds on grasses when available. Its morphology and behaviour is, similar to that of *E*. *africanus*, adapted to arid environments [136]. *Equus hydruntinus* is similar in morphology to *E*. *hemionus* [138] and, hence, was most likely also similar in ecology and behaviour [138]. It is thus likely that *E*. *ferus* will be able to coexist with these species of *Equus* currently living within the native range of *E*. *ferus*, and rewilding with *E*. *ferus* in these regions should hence not be problematic from this perspective. Therefore, there are no zoogeographic reasons against rewilding with *E*. *ferus* in the Americas, Eurasia and northern Africa.

There are large areas of suitable habitat for *E*. *ferus* in sub-Saharan Africa, Australia and New Zealand. However, these areas are less obviously relevant for rewilding with this species. *E*. *ferus* is not native to any of the areas. The lack of *E*. *ferus* as a native species in sub-Saharan Africa is most likely due to the presence of zebras (*E*. *quagga*, *E*. *zebra* and *E*. *grevyi*), which are very similar to *E*. *ferus* in ecology and social behaviour [142], thereby likely excluding *E*. *ferus* from this region. Their striped skin confers advantages in regions with tsetse flies, as the horizontal stripes might prevent attraction of tsetse flies, thereby avoiding infection by trypanosomiasis, which is transferred by the tsetse fly [143]. Hence, rewilding with *E*. *ferus* is not generally relevant or desirable in the zebra-occupied parts of sub-Saharan Africa, although negative effects on non-equid species are unlikely given the long equid history in the region [41]. The native mammal fauna of Australia is very different from other continents; notably, all larger native terrestrial mammals are marsupials, and the region has never been occupied by equids or species similar to equids. Here, feral *E*. *ferus* are usually considered pests with large impacts on agriculture and native habitats. The effects on the native fauna and vegetation are less clear, however, as both positive and negative effects on native wildlife and flora have been reported [48, 144]. The situation is similar in New Zealand but even more extreme as the region did not naturally harbour terrestrial mammalian herbivores. Introduced mammalian herbivores are generally perceived to be highly damaging there [The Department of Conservation, New Zealand], and feral *E*. *ferus* have threatened vulnerable native plant species [50].

### Habitat suitability within five major rewilding areas in Europe

At the landscape scale, all five large rewilding areas in Europe had suitable climates for wild-living horses according to the MAXENT and CEM models (Fig 7). They also all contained substantial primary habitat with interconnecting secondary habitat away from cropland and urban environment, reducing the risk of conflicts with humans (Fig 7). Ideally, rewilding introductions should produce populations able to maintain themselves without human intervention. For animals, it is important to ensure that the area can sustain the minimum viable population of the species. The minimum viable population (MVP) size for feral *E*. *ferus* has been estimated to be 72–300 individuals [51, 145]. Home ranges of breeding bands of wild-living *E*. *ferus* vary in size from 1–48 km^2^ dependent on the availability of forage and water [17, 67, 72, 141]. Band sizes of feral *E*. *ferus* vary between 2–20 individuals but are often 4 individuals [141]. Assuming a band-size of 4 individuals, a minimum and maximum band home range of 1 and 48 km^2^, respectively, and a minimum and maximum MVP of 72 and 300, the total area needed to sustain a viable population of *E*. *ferus* might be between 18–3,600 km^2^ (1,800–360,000 ha), depending on the quality of the habitat. Currently, 800–1000 *E*. *ferus* live without human intervention in the 6000-ha Oostervaardersplassen in the Netherlands [137]. The rewilding areas in Western Iberia, the Danube delta, Velebit, Eastern Carpathians and Southern Carpathians cover 1,750,000 ha, 633,000 ha, 249,000 ha, 208,000 ha and 226,000 ha, respectively, and include approximately 1,150,000 ha, 329,000 ha, 47,000 ha, 42,000 ha and 86,000 ha of primary habitat, respectively. Hence, these areas are all large enough to sustain viable populations of *E*. *ferus*. However, depending on factors such as habitat quality and genetic diversity, much smaller areas may be sufficient to sustain populations for long periods of time. In the report on rewilding with horses by Rewilding Europe, the minimum area requirement suggested is from at least 450 ha in a fertile delta to 4,500 on poor soil [17]. Several feral populations exist on small islands and have done so for decades or centuries, e.g., Tærø in Denmark (171 ha) [8989] and Shackleford Banks (approx. 1200 ha) [146].

### General issues to consider for rewilding with horses

The widespread occurrence of suitable climates and habitats within the very large former range of *E*. *ferus* together with its important functions as a grazer causes wild-living horses to be a key candidate for rewilding in large parts of the world. To re-establish wild-living or semi-wild horses as a component in rewilding-based management of natural areas and broader landscapes will require consideration of additional ecological issues beyond biogeography and habitat area as well as the complexity of human-horse relations. One issue to consider is the role of predation. Although we do not have a clear understanding of the extent to which horse populations naturally were top-down limited by predation, they were certainly sometimes important prey for large carnivores [147]. Further, feral populations may sometimes be limited by predation and may also sometimes increase rapidly in the absence of predators [148, 149], thereby greatly impacting vegetation [150, 151] and, in some cases, having negative effects on other biodiversity [152]. There are as yet no definite answers to this issue. Clearly, more research is needed to better understand the potential role of top-down regulation in the case of wild-living horses. In some real-world cases, it may be feasible to also reintroduce relevant predators. Further, the recovery of large carnivores in some regions, such as Europe [153], should also allow re-establishment of natural predator-prey interactions. More generally, rewilding projects will be open-ended restoration projects with a focus on restoring natural processes and should be ongoing monitoring and evaluation of their ecological dynamics [154], allowing intervention if it becomes necessary. Horses have a long-standing and complex relation to humans and society, which may in some cases complicate their use in rewilding and require careful attention. For example, in many countries, horses are classified as livestock and thus fall under legislation regarding management of livestock. In practice, this means that laws regarding management of livestock often demand fencing, provision of food, monitoring, veterinary care and shelter, making establishment of wild-living populations difficult. In the Netherlands, however, the judicial system has classified the feral horses in Oostvaardersplassen as wild animals that no longer fall under the laws for livestock [137]. There may also be conflicts with ranchers and other agriculturalists as wild-living *E*. *ferus* and domestic livestock may compete for forage or there may be a risk of disease transmission between wild-living horses and livestock [48, 67, 155]. Public views on feral horses range from a pest species to an iconic wild animal that deserve protection [155, 156], which may render management more difficult. Notably, human control of feral horse populations is often controversial as the public may perceive management actions such as culling of excess individuals to be animal cruelty [144].

## Conclusions

Our species distribution modelling results provide a conservative estimate of areas with suitable climates for wild-living horses, given the human-constrained distribution of feral populations and, with additional information on habitat selection and historical distribution, allow us to identify areas suitable for rewilding with horses. This study demonstrates that feral populations of *E*. *ferus* occur under a wide variety of climatic conditions, from cold boreal to warm tropical climates. As a result, large areas of the world could potentially harbour feral horse populations, even when restricting these to ≥100-km^2^ areas dominated by open and semi-open natural habitats. Notably, essentially throughout the wide Late Pleistocene range of *E*. *ferus*, there are still today many areas that would be suitable for wild-living horse populations, indicating that current feral populations have retained all or most of the species’ former wide ecological adaptability. Hence, *E*. *ferus* is an obvious species to use in rewilding in much of the world [17] due to its former very wide range, its wide extant ecological tolerance, its particular role as a grazer and our extensive knowledge of its ecology, behaviour and management.

## Supporting Information

S1 Fig Comparison of boxplots for the focal climatic variables for the full and thinned datasets. Boxplots of Mean temperature in the coldest quarter (MTCQ) (**A**), precipitation in the driest quarter (PDQ) (**B**) and precipitation in the coldest quarter (PCQ) (**C**) for the full (n = 186) (white) and the thinned (n = 76) (grey) datasets. Whiskers mark the 1^st^ and 3^rd^ quartiles, and the line indicates the median. Dots are outliers.
(TIF)

S2 Fig Comparison of ensemble maps for the focal climatic variables for the full and thinned datasets. Distributions of suitable climate for *E*. *ferus* according to the two best models: M2: Mean temperature in the coldest quarter (MTCQ) and precipitation in the driest quarter (PDQ); and M3: MTCQ, PDQ and precipitation in the coldest quarter (PCQ) built on the thinned dataset (n = 76) (**A**) and the full dataset (n = 186) (**B**). Colours indicate the number of overlapping models.
(TIF)

S3 Fig Comparison of MTCQ and MTDQ. Distributions of suitable climates for *E*. *ferus* according to M3, mean temperature in the coldest quarter (MTCQ), precipitation in the driest quarter (PDQ) and precipitation in the coldest quarter (PCQ) (dark green), and the model composed of mean temperature in the driest quarter (MTDQ), PCQ and PDQ (light green). The figure shows the overlap in the distributions of the two models (medium green). Both models were constructed with a threshold of a minimum 10% training presence and 50% test data.
(TIF)

S4 Fig Past distribution of horses (*E*. *ferus*). Latest Pleistocene-early Holocene (ca. 10.000 to 3500 BC) (modified from [25]) and mid/late Holocene (3500 BC-present) distributions of *E*. *ferus*.
(TIF)

S5 Fig The current distribution of primary habitat for horses (*E*. *ferus*) and extant equids. The distribution of the primary habitat within (dark green) and outside (Brown) the native range of *E*. *ferus* at a 10-km resolution within the extent of the final MAXENT models (**A**). The historical distribution of *E*. *africanus* (**B**) and the three extant species of zebra: *Equus grevyi; Equus quagga* and *Equus zebra (*formerly *E*. *burchelli)* (**C**), the current distribution of *E*. *kiang* and *E*. *hemionus* (**D**) **B-D** from [41].
(TIF)

S1 Table Main sources for information on wild-living populations of *E*. *ferus* worldwide. (DOCX)

S2 Table Coordinates and climatic variables for the full and thinned datasets. Thinned dataset in bold. Feral populations denoted by F, and Semiferal populations denoted by S.
(XLSX)

S3 Table Pearson’s correlation between all variables considered for modelling the distribution of wild-living *E*. *ferus*. (DOCX)

S4 Table GlobCover land use categories used as primary and secondary *E*. *ferus* habitat. (DOCX)
