# Supplementary figures and images for: A Geographic Assessment of the Global Scope for Rewilding with Wild-Living Horses (Equus ferus)
Source: PLoS One. 2015 Jul 15;10(7):e0132359. doi: 10.1371/journal.pone.0132359 (PMC4503665; doi:10.1371/journal.pone.0132359)

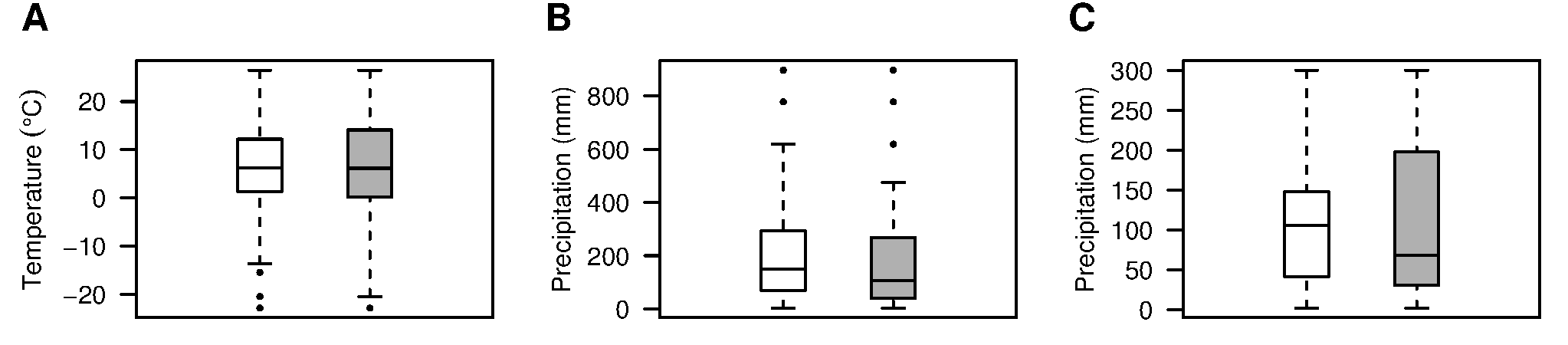

Supplement: S1 Fig — Boxplots of Mean temperature in the coldest quarter (MTCQ) (A), precipitation in the driest quarter (PDQ) (B) and precipitation in the coldest quarter (PCQ) (C) for the full (n = 186) (white) and the thinned (n = 76) (grey) datasets. Whiskers mark the 1st and 3rd quartiles, and the line indicates the median. Dots are outliers. (TIF) [file pone.0132359.s001.tif]

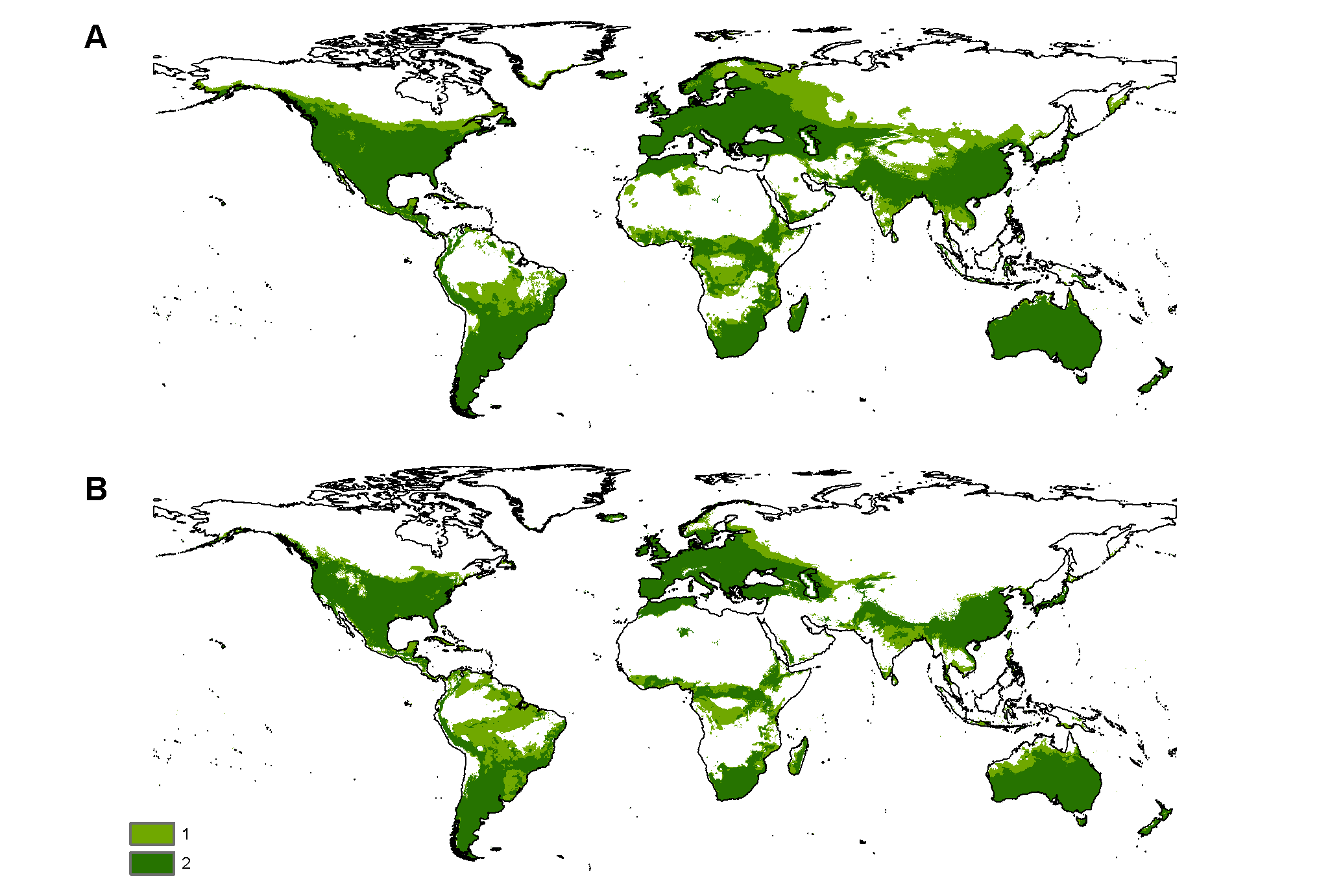

Supplement: S2 Fig — Distributions of suitable climate for E. ferus according to the two best models: M2: Mean temperature in the coldest quarter (MTCQ) and precipitation in the driest quarter (PDQ); and M3: MTCQ, PDQ and precipitation in the coldest quarter (PCQ) built on the thinned dataset (n = 76) (A) and the full dataset (n = 186) (B). Colours indicate the number of overlapping models. (TIF) [file pone.0132359.s002.tif]

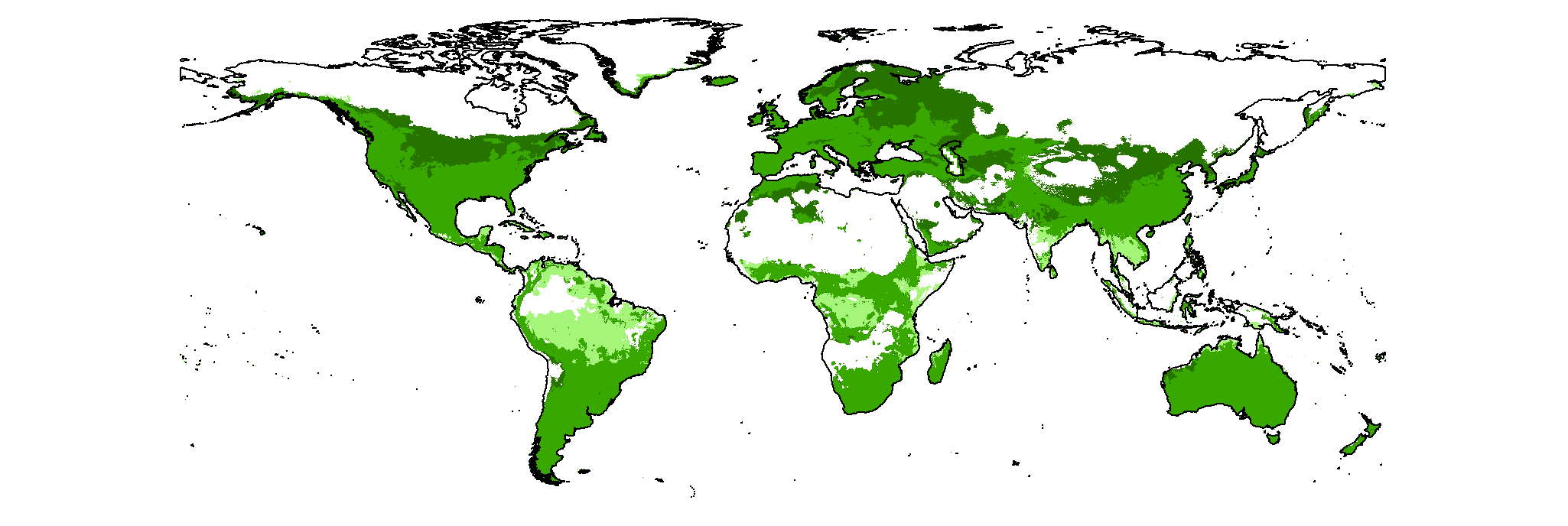

Supplement: S3 Fig — Distributions of suitable climates for E. ferus according to M3, mean temperature in the coldest quarter (MTCQ), precipitation in the driest quarter (PDQ) and precipitation in the coldest quarter (PCQ) (dark green), and the model composed of mean temperature in the driest quarter (MTDQ), PCQ and PDQ (light green). The figure shows the overlap in the distributions of the two models (medium green). Both models were constructed with a threshold of a minimum 10% training presence and 50% test data. (TIF) [file pone.0132359.s003.tif]

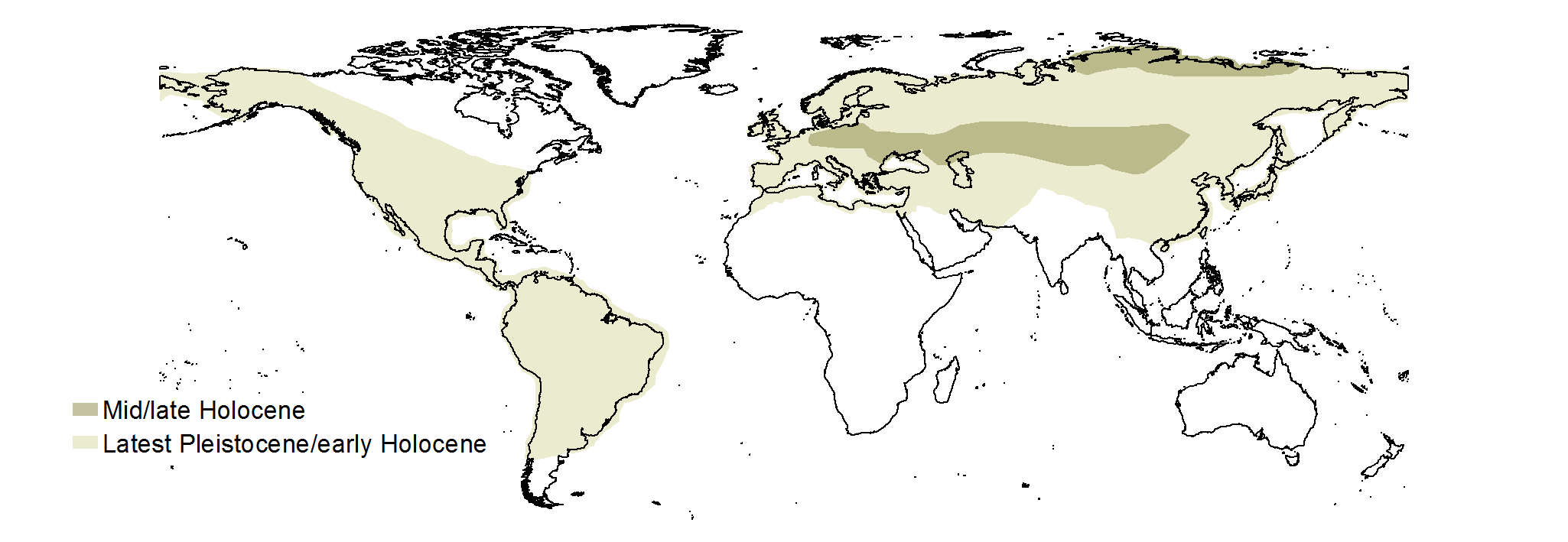

Supplement: S4 Fig — Latest Pleistocene-early Holocene (ca. 10.000 to 3500 BC) (modified from [25]) and mid/late Holocene (3500 BC-present) distributions of E. ferus. (TIF) [file pone.0132359.s004.tif]

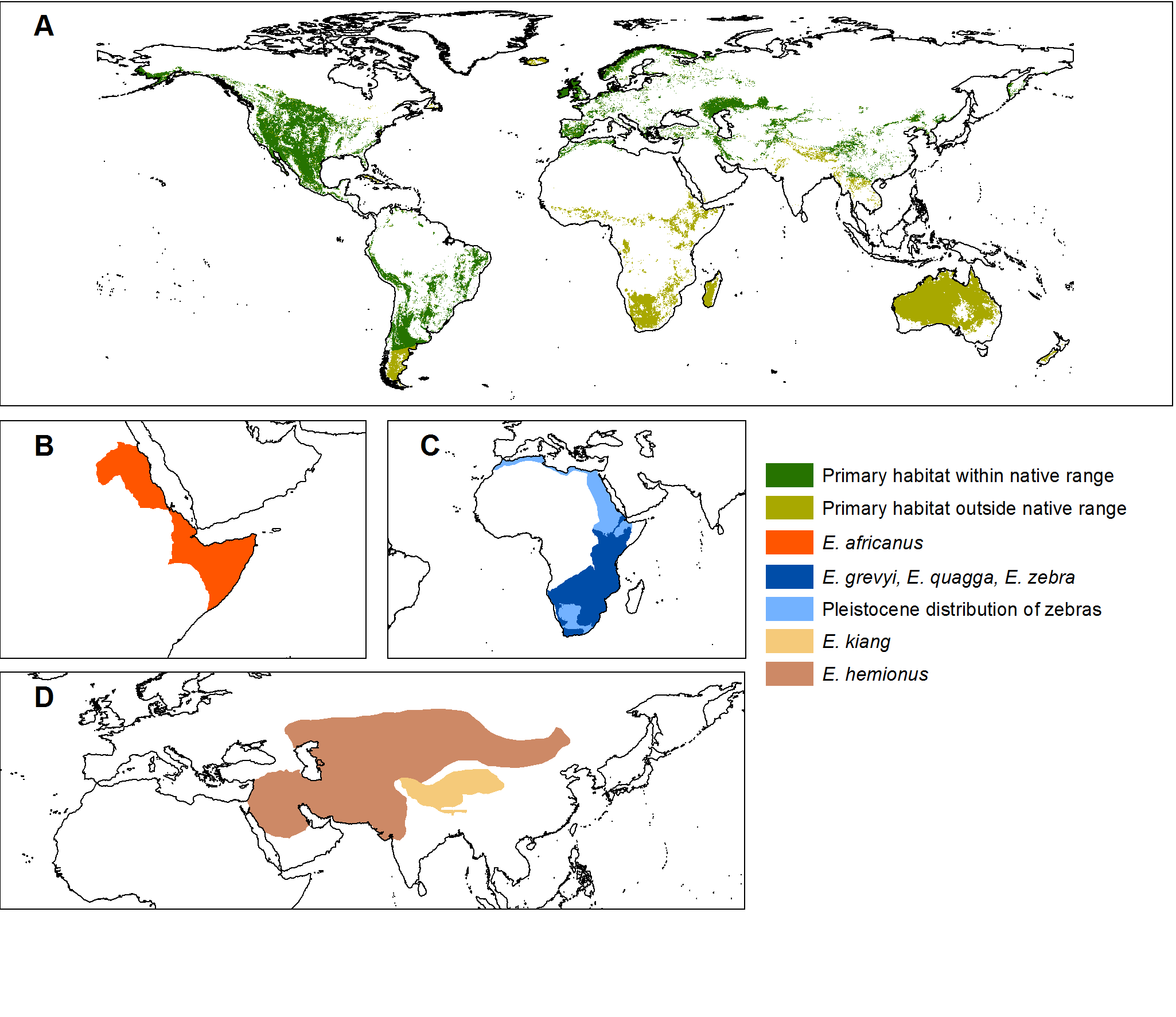

Supplement: S5 Fig — The distribution of the primary habitat within (dark green) and outside (Brown) the native range of E. ferus at a 10-km resolution within the extent of the final MAXENT models (A). The historical distribution of E. africanus (B) and the three extant species of zebra: Equus grevyi; Equus quagga and Equus zebra (formerly E. burchelli) (C), the current distribution of E. kiang and E. hemionus (D) B-D from [41]. (TIF) [file pone.0132359.s005.tif]
