# Supplementary material for: A Geographic Assessment of the Global Scope for Rewilding with Wild-Living Horses (Equus ferus)
Source: PLoS One. 2015 Jul 15;10(7):e0132359. doi: 10.1371/journal.pone.0132359 (PMC4503665; doi:10.1371/journal.pone.0132359)
Supplement: S1 Table — (DOCX) [file pone.0132359.s006.docx]

| **Region** | **Main source** |
| --- | --- |
| **Africa** |  |
| *Literature* | Greyling T (2005) Factors affecting possible management strategies for the Namib feral horses. Ph.D. Thesis, The North-West University. Available: <http://dspace.nwu.ac.za/handle/10394/1244>. Accessed 16 September 2013. |
| *Governmental agencies and private organizations* | Biosphere reserve Kogelberg |
| *Personal communication* | Telane Greyling |
| **Asia** |  |
| *Literature* | Goonatilake, S. de A., Ekanayake, S., Kumara, P. B. Terney Pradeep, Liyanapathirana, D., Weerakoon, D.K., and Wadugodapitiya, A. 2013. Sustainable Development of Delft Island: An ecological, socio-economic and archaeological assessment. International Union for Conservation of Nature, Colombo, Sri Lanka & Government of Sri Lanka. viii + 86 pp |
| *Governmental agencies and private organizations* | Dibrusaikhowa National Park |
| *Personal communication* | Ashraf M. Kahlil |
| **Europe** |  |
| *Literature* | Prishutova ZG (2010) Feral horses (*Equus caballus*) as a component of protected steppe ecosystems in the Rostovskii Nature Reserve. Russian Journal of Ecology 41: 55-59.  Lamoot I, Meert C and Hoffmann M (2005) Habitat use of ponies and cattle foraging together in a coastal dune area. Biological Conservation 122:523-536. |
| *Governmental agencies and private organizations* | Brecon Beacon National Park  Roztocze National Park  Dartmoor National Park  Exmoor National Park  New Forest National Park  New Forest National Park  Snowdonia National Park  Pembrokeshire Coast National Park  Connemara Pony Breeders Society  Doñana Biological Station  Natural England  The Asturcon Pony Breeding Association (ACPRA)  Parc naturel regional de Camargue  Pasaules Dabas Fonds  Save Foundation  Statsbosbeheer (The Nature Agency of Holland)  Suffolk Wildlife Trust  The Danish Nature Agency, The Danish Ministry of Environment  The RSPB  Westhoek National reserve  www.wildpferde.de |
| *Personal communication* | Laura Lagos, Jesus Ma Martinez Sáiz, Klaus M. Scheibe |
| **New Zealand and Australia** |  |
| *Literature* | Dawson MJ, Lane C, Saunders G. Proceedings of the national feral horse management workshop; 2006; Canberra |
| *Governmental agencies* | Department of Conservation, New Zealand |
|  | Department of Sustainability, Environment, Water, Population and Communities, Australia |
| **North America** |  |
| *Literature* | Rheinhardt RD, Rheinhardt MC (2004) Feral horse seasonal habitat use on a coastal barrier split. Journal of Range Management 57: 253-258.  Bhattacharyya J, Slocombe DS, Murphy SD (2011) The “Wild” or “Feral” Distraction: Effects of Cultural Understandings on Management Controversy Over Free-Ranging Horses (*Equus ferus caballus*) Human Ecology 39: 613-625.  Contasti AL, Tissier EJ, Johnstone JF, McLoughlin PD (2012) Explaining Spatial Heterogeneity in Population Dynamics and Genetics from Spatial Variation in Resources for a Large Herbivore. Plos One 7: e47858 |
| *Governmental agencies and private organizations* | BLM (Bureau of Land Management, US)  Chincoteague Volunteer Fire Department.  Environment and Sustainable Resource Development  National Park Service USA  Saskatchewan Parks |
| **South America** |  |
| *Literature* | Scorolli AL, Lopez Cazorla AC, Tejera LA (2006) Unusual mass mortality of feral horses during a violent rainstorm in Parque Årovincial Tornquist, Argentina. Mastozoología Neotropical 13: 255-258.  Scorolli AL, Lopez Cazorla AC (2010) Feral horse social stability in Tornquist Park, Argentina. Mastozoologia Neotropical 17: 391-396.  Perez A, Garcia ME, Quijada J, Aguirre A, Cartana ML, Armas S (2010) Strongyles Parasitism in Wild Venezuelan Horses from Hato El Frio, State of Apure, Venezuela a Preliminary Study. Revista Cientifica-Facultad De Ciencias Veterinarias 20: 32-36. |
| *Personal communication* | Alberto Scorolli and Hugo Lopez-Arevalo |
