# Supplementary material for: A Geographic Assessment of the Global Scope for Rewilding with Wild-Living Horses (Equus ferus)
Source: PLoS One. 2015 Jul 15;10(7):e0132359. doi: 10.1371/journal.pone.0132359 (PMC4503665; doi:10.1371/journal.pone.0132359)
Supplement: S3 Table — (DOCX) [file pone.0132359.s008.docx]

|  | MTWeQ | MTDQ | MTWQ | MTCQ | AP | PWQ | PCQ | PWeQ | PDQ |
| --- | --- | --- | --- | --- | --- | --- | --- | --- | --- |
| MTDQ | 0.240 |  |  |  |  |  |  |  |  |
| MTWQ | **0.871** | 0.500 |  |  |  |  |  |  |  |
| MTCQ | 0.595 | **0.817** | **0.682** |  |  |  |  |  |  |
| AP | 0.094 | 0.355 | 0.079 | 0.441 |  |  |  |  |  |
| PWQ | 0.309 | 0.199 | 0.225 | 0.384 | **0.816** |  |  |  |  |
| PCQ | -0.235 | 0.303 | -0.177 | 0.205 | 0.572 | 0.065 |  |  |  |
| PWeQ | 0.286 | 0.429 | 0.288 | 0.587 | **0.916** | **0.849** | 0.357 |  |  |
| PDQ | -0.343 | -0.003 | -0.394 | -0.118 | 0.555 | 0.327 | 0.583 | 0.218 |  |
| HII | -0.137 | 0.104 | -0.028 | 0.044 | 0.327 | 0.226 | 0.305 | 0.265 | 0.233 |

Correlated variables (Pearson’s r > 0.650) are shown in bold.
