# Supplementary material for: A Geographic Assessment of the Global Scope for Rewilding with Wild-Living Horses (Equus ferus)
Source: PLoS One. 2015 Jul 15;10(7):e0132359. doi: 10.1371/journal.pone.0132359 (PMC4503665; doi:10.1371/journal.pone.0132359)
Supplement: S4 Table — (DOCX) [file pone.0132359.s009.docx]

| **Habitat type** | **Legend** | **Description** |
| --- | --- | --- |
| Primary | 110 | Mosaic forest or shrubland (50-70%) / grassland (20-50%) |
|  | 120 | Mosaic grassland (50-70%) / forest or shrubland (20-50%) |
|  | 130 | Closed to open (>15%) (broadleaved or needleleaved, evergreen or deciduous) shrubland (<5m) |
|  | 140 | Closed to open (>15%) herbaceous vegetation (grassland, savannas or lichens/mosses) |
|  | 150 | Sparse (<15%) vegetation |
|  | 180 | Closed to open (>15%) grassland or woody vegetation on regularly flooded or waterlogged soil - Fresh, brackish or saline water |
| Secondary | 40 | Closed to open (>15%) broadleaved evergreen or semi-deciduous forest (>5m) |
|  | 60 | Open (15-40%) broadleaved deciduous forest/woodland (>5m) |
|  | 90 | Open (15-40%) needleleaved deciduous or evergreen forest (>5m) |
|  | 100 | Closed to open (>15%) mixed broadleaved and needleleaved forest (>5m) |
|  | 160 | Closed to open (>15%) broadleaved forest regularly flooded (semi-permanently or temporarily) - Fresh or brackish water |
